# Influence of Sagittal Cervical and Thoracic Range of Motion on Neck Pain Severity in Young White-Collar Workers: A Cross-Sectional Study

**DOI:** 10.3390/jcm13185412

**Published:** 2024-09-12

**Authors:** Tomasz Kuligowski, Anna Skrzek, Błażej Cieślik

**Affiliations:** 1 Faculty of Physiotherapy, Wroclaw University of Health and Sport Sciences, 51-612 Wroclaw, Poland; anna.skrzek@awf.wroc.pl; 2 Healthcare Innovation Technology Lab, IRCCS San Camillo Hospital, 30126 Venice, Italy; blazej.cieslik@hsancamillo.it

**Keywords:** neck pain, cervical spine, thoracic spine, range of motion, occupational diseases

## Abstract

**Background**: Neck pain (NP) is a prevalent musculoskeletal disorder, especially among individuals with sedentary occupations. The interplay between cervical and thoracic spine mobility is hypothesized to contribute significantly to NP severity, yet this relationship requires further exploration. **Methods**: This cross-sectional study involved 179 young white-collar workers with NP lasting for at least six weeks. Participants were stratified into mild (*n* = 78) and moderate (*n* = 101) pain groups based on their scores on the Northwick Park Neck Pain Questionnaire (NPQ). Cervical and thoracic range of motion (ROM) in the sagittal plane was measured using inclinometers. NP severity was further assessed using the NPQ and the Neck Disability Index (NDI). Correlation, regression, and mediation analyses were conducted to investigate the relationship between cervical and thoracic ROM and NP severity. **Results**: Thoracic ROM was higher in the mild pain group (median: 47.35, IQR: 10.13) than in the moderate pain group (median: 42.10, IQR: 13.60; *p* < 0.001). The NDI had a negative correlation with thoracic ROM (*r* = −0.65; *p* < 0.05) and a positive correlation with cervical ROM (*r* = 0.84; *p* < 0.01). Additionally, thoracic ROM mediated the effect of cervical ROM on NP, particularly influencing NDI scores (*p* < 0.01). **Conclusions**: This study found a significant association between reduced thoracic ROM and increased NP severity, highlighting the role of thoracic spine mobility in NP among young white-collar workers. Targeted interventions for thoracic dysfunction may reduce compensatory cervical strain and improve NP management, suggesting that thoracic spine assessments should be integrated into routine clinical evaluations.

## 1. Introduction

Neck pain (NP) can have multifactorial backgrounds. It is the second most common musculoskeletal disorder in modern society, with a prevalence of about 2.7% in adults. It leads to prolonged work absence and is more likely to occur in people with sedentary jobs, mostly females in their fifth and sixth life decades [1].

Neck pain is a broad term. It usually covers typical overload, muscle-related symptoms, headaches, radicular pain, discogenic pain, discopathy, facet joint-related disorders, and others. Due to this fact, the NP term should be used in the literature with caution [2,3]. In this paper, it is suggested to use a simple division of the NP to “*neck pain*”, where the main symptoms are localized in the cervical area, and “*neck-related pain*” to all those pathologies where the pain occurrence can be peripheral with its source within the cervical region. 

The etiology and manifestations of cervical pain are multifaceted yet interconnected through mechanisms of a self-perpetuating cycle. Current concepts about the main risk factors include biological and psychological types with subtypes. Within the musculoskeletal area, the biomechanical aspects are considered the leading ones, especially in young adults where a biomechanical, long-term overload background is primarily present [4,5,6,7,8]. Neck pain symptoms, when viewed through the lens of biomechanics, often stem from the complex interplay between the cervical spine’s structure and the forces acting upon it. The cervical spine, composed of seven vertebrae and associated muscles, ligaments, and intervertebral discs, supports the head’s weight. This system allows for a wide range of motion, including flexion, extension, rotation, and lateral bending. However, this mobility comes at the cost of stability, making the neck particularly vulnerable to biomechanical stress [9,10,11]. Poor posture, such as forward head posture commonly seen in people who spend long hours on computers or mobile devices, can lead to an imbalance where the head’s weight exerts excessive force on the cervical spine [12,13,14,15]. This can cause muscle imbalance and weakness in the primary active stabilizers, especially longus colli with other flexors [16]. Afterwards, an improper segmental range of motion can be observed, leading to cervical disc disorders [17,18]. 

In some cases, nerve impingement can occur, leading to sharp, radiating pain, tingling, or numbness in the arms and hands and further neurological deficits. Muscle imbalances, such as tightness in the upper trapezius and levator scapulae muscles, are common, further exacerbating pain and discomfort. Biomechanical neck pain can also lead to secondary symptoms such as headaches [19,20]. Understanding the biomechanical causes of neck pain is crucial for developing effective treatment strategies, which often include correcting posture, strengthening weak muscles, and improving overall spinal alignment through targeted exercises and physical therapy. Other cases might need surgical treatment; however, most are treated conservatively [21]. 

Non-surgical treatment includes, among others, hands-on techniques, therapeutic exercises, and muscle activation programs. The literature describes those widely [22,23,24,25,26]. Authors agree that none of the methods is significantly superior to others when applied alone, and as a result, multimodal forms of management are mainly prescribed [27]. One of the concepts assumes that neck pain can result from cervicothoracic range of motion (ROM) imbalance. Biomechanically, as observed in other human body areas, the decreased ROM in one joint will likely force increased ROM in adjacent [28,29]. In the following, some authors observed positive results of mobilization and manipulation techniques aimed at the thoracic spine in neck pain individuals [30,31,32]. The literature, however, needs to include more in the field of explaining this relationship and its range, especially in young individuals.

Thus, the primary aims of this study were to assess the range of motion (ROM) of the cervical and thoracic spine in the sagittal plane in young office workers with mild to moderate neck pain disability, investigate the association between neck pain levels and cervical and thoracic ROM, and explore the potential mediating role of cervical and thoracic total ROM on neck pain variables. We hypothesize that thoracic mobility is critical in preventing compensatory cervical hypermobility and neck pain in young white-collar workers.

## 2. Materials and Methods

### 2.1. Participants and Study Design

This study was designed as a cross-sectional study with convenience sampling, prepared following the STROBE guidelines, and conducted between January and May 2022 at a private physiotherapy clinic (“*Klinika Fizjoterapii*”) in Wrocław, Poland [33]. This study was conducted in accordance with the Declaration of Helsinki, and the study protocol received prior approval from the Institutional Review Board (IRB) of the Wroclaw University of Health and Sport Sciences (11/2015). Prior to the commencement of the research, all subjects were provided with information regarding the study procedures and their right to decline or withdraw at any time. Informed consent was obtained from all participants before their study enrollment.

The inclusion criteria were young adult white-collar workers aged between 22 and 35 years who reported neck pain lasting for at least six weeks, with a minimum intensity of 20 points on the Northwick Park Neck Pain Questionnaire (NPQ), resulting in subjective disability in activities of daily living. All volunteers were seeking help in a private physiotherapy clinic and had not had any undergoing treatment. Based on NPQ results, the patients were categorized into the group with mild (NPQ score of 20–39) and moderate (NPQ score of 40–59) neck pain disability. Exclusion criteria consisted of recent severe neck trauma (whiplash or other within the last six months), history of neck or spinal surgery, diagnosed neurological disorders affecting the cervical spine (cervical myelopathy, radiculopathy, multiple sclerosis), and other chronic pain conditions that might interfere with the assessment of neck pain (e.g., fibromyalgia, significant unilateral muscle strength deficits, significant scoliosis, congenital cervical–thoracic region disorders, antidepressant intake, or rheumatologic undergoing treatment). 

### 2.2. Outcome Measures and Procedures

This study used a structured, custom-made questionnaire to gather information on demographics (sex, age, body mass, and height), working experience, self-reported exercise level, desk regulation, and subjective neck pain assessment.

#### 2.2.1. Patient Self-Reported Outcome Measure

Subjective neck pain assessment was conducted using the Northwick Park Neck Pain Questionnaire (NPQ) and the Neck Disability Index (NDI). Both questionnaires are self-reported tools used to assess the severity and impact of neck pain on daily activities. The scores from these questionnaires are summed and converted to a percentage, providing a standardized measure of neck pain-related disability. While both tools are very similar, using the NDI and the NPQ together in one study allows for a comprehensive evaluation of neck pain. The 10-item NDI focuses more on specific functional limitations, such as personal care, lifting, and headaches [34], whereas the 9-item NPQ captures broader aspects of daily activities, overall pain impact, and the effect on work and recreation [35]. This dual approach ensures a more thorough assessment of patient disability, covering detailed functional impairments and general daily activity limitations.

#### 2.2.2. Range of Motion Assessment

Two inclinometers (12-1056, Baseline^®^, Fabrication Enterprises, White Plains, NY, USA) were used to assess the range of motion (ROM) of the cervical and thoracic spine in the sagittal plane (flexion and extension). Measurements for flexion and extension were taken three times, and their average value was used for further analysis. The range of flexion and extension was summed to calculate the total ROM. All the procedures followed American Medical Association (AMA) guidelines and were supported by the literature [36,37,38]. The neck and thoracic range of motion (ROM) were measured as active (AROM). The patient was sitting while measuring cervical ROM and standing during the measurement of thoracic ROM. A single inclinometer was placed at the top of the patient’s head to measure cervical ROM. In contrast, two inclinometers (one at T1 and the other at T12 vertebrae) were used simultaneously to measure thoracic ROM. After placing the inclinometers, the participant was asked to perform slow but full flexion and return to the starting position. The same task was repeated for extension and performed three times for each area. While assessing the thoracic ROM, the patient was asked to bend forward towards the floor and return to the starting position. The analogic move was performed to achieve full extension. Thoracic ROM was calculated by subtracting the value at the T12 from the value at the T1.

### 2.3. Data Analysis

The data analysis used JASP version 0.16.4 (University of Amsterdam, Amsterdam, The Netherlands). Means and standard deviations (SDs) were calculated for demographic characteristics and work experience (years). At the same time, quantities and percentages were reported for categorical data such as BMI distribution and self-reported physical activity levels. The Kolmogorov–Smirnov test indicated a non-normal distribution for NPQ, NDI, and cervical and thoracic ROM in both groups. Therefore, the Mann–Whitney U test was used to compare continuous variables between groups, and chi-square (*χ*^2^) tests were applied to compare categorical data. Stepwise multiple linear regression was used to identify the association between pain level (NPQ and NDI) and cervical and thoracic total ROM. Prior to conducting the regression analysis, the monotonic relationship between the outcome variable and the independent variables was assessed using scatterplots and Spearman’s rho test. Next, the normality of residuals was evaluated using Q-Q plots, and the absence of multicollinearity was confirmed using the variance inflation factor (VIF). Finally, mediators were tested by calculating bias-corrected 95% confidence intervals (CIs) using bootstrapping (5000 samples) with JASP software. Two mediation pathways were examined: (1) thoracic total ROM as a mediator of the effect of cervical ROM on pain variables (NPQ and NDI), and (2) cervical total ROM as a mediator of the effect of thoracic ROM on pain variables. The results were presented as the total, direct, and indirect effect sizes. The significance level (*α*) was set at 0.05 to determine statistical significance.

To verify the reliability of our study’s findings, we conducted a post-hoc power analysis using G*Power 3.1.9.6 software from Heinrich-Heine-University Düsseldorf, Germany. The primary outcomes analyzed were cervical and thoracic total ROM scores. The analysis included effect sizes (Cohen’s *d*) of −0.48 for cervical total ROM and 0.72 for thoracic total ROM, with an alpha level of 0.05, logistic parent distribution, and sample sizes of 78 and 101, respectively. This analysis resulted in statistical power values of 0.91 for cervical ROM and 0.99 for thoracic ROM. These high-power levels indicate a strong likelihood of detecting the expected effects, which supports the reliability of our study’s findings.

## 3. Results

### 3.1. Participant Characteristics

This study included a total of 179 participants, of which 139 (77.65%) were women. Participants were categorized into groups based on their reported pain levels: mild pain (*n* = 78, 43.58%) and moderate pain (*n* = 101, 56.42%). Table 1 illustrates the participants characteristics. The average age of the participants was 30.17 years (SD: 3.33), with no significant difference between the mild pain group and the moderate pain group (*p* = 0.69). In terms of body mass and BMI, the moderate pain group had a significantly higher average body mass compared to the mild pain group, with *p*-values of 0.02 and 0.003, respectively. Participants’ working experience averaged 7.03 years (SD: 3.55), with those in the moderate pain group having significantly more experience (mean: 7.77 years, SD: 3.53) than those in the mild pain group (mean: 6.08 years, SD: 3.37; *p* < 0.01). Desk regulation was reported by 62.82% of participants in the mild pain group and 72.28% in the moderate pain group (*p* = 0.18). Self-reported exercise levels varied significantly between groups (*p* = 0.008).

### 3.2. Clinical Variables by Pain Intensity

The groups differed significantly in pain and cervical and thoracic range of motion. For the NPQ, the median score for the mild pain group was 37.50 [IQR: 5.60], while the moderate pain group had a significantly higher median score of 47.20 [IQR: 8.40] (*p* < 0.001). The NDI showed a median of 7.00 [IQR: 8.00] in the mild pain group and 11.00 [IQR: 11.00] in the moderate pain group, indicating a significant difference (*p* < 0.001).

**Figure 1:**
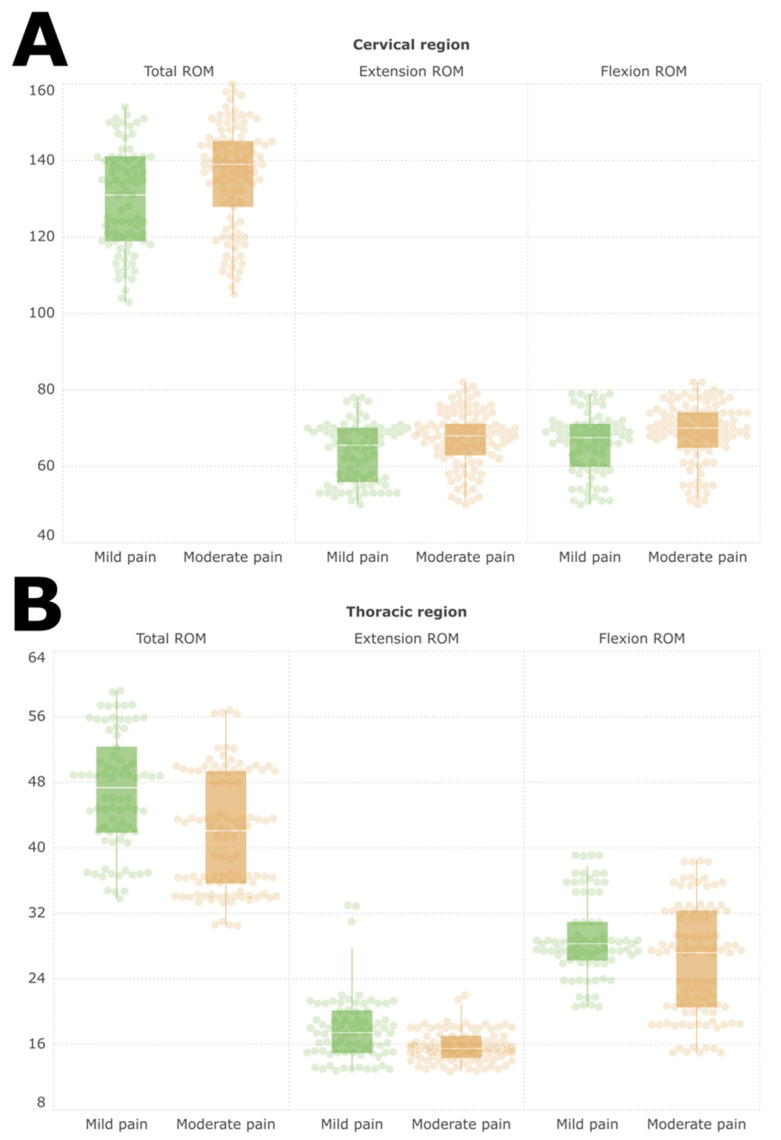
Cervical (**A**) and thoracic (**B**) region of the spine ROM within each subgroup.

Figure 1 illustrates each subgroup’s cervical and thoracic region of the spine ROM. Cervical total ROM was significantly different between the groups, with the mild pain group having a median of 131.00 [IQR: 21.75] and the moderate pain group having a median of 139.00 [IQR: 17.00] (*p* = 0.002). Specifically, cervical flexion had a median of 67.50 [IQR: 11.00] in the mild pain group and 70.00 [IQR: 9.00] in the moderate pain group (*p* = 0.007). Cervical extension had medians of 65.50 [IQR: 14.00] and 68.00 [IQR: 8.00] in the mild and moderate pain groups, respectively (*p* = 0.007). Thoracic total ROM was significantly higher in the mild pain group, with a median of 47.35 [IQR: 10.13] compared to 42.10 [IQR: 13.60] in the moderate pain group (*p* < 0.001). Thoracic flexion had a median of 28.30 [IQR: 4.60] in the mild pain group and 27.20 [IQR: 11.70] in the moderate pain group (*p* = 0.004). Thoracic extension showed a median of 17.45 [IQR: 5.05] in the mild pain group, which was significantly higher than the median of 15.50 [IQR: 2.60] in the moderate pain group (*p* < 0.001).

### 3.3. Correlation, Regression, and Mediation Analysis Results

**Figure 2:**
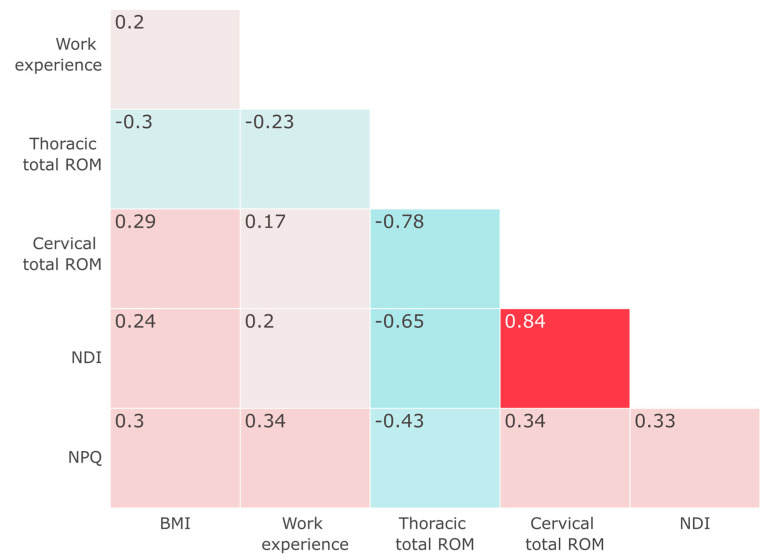
Correlation heatmap. Note: All displayed correlation values are statistically significant at *p* < 0.05.

Figure 2 illustrates the correlation heatmap of the variables. The NPQ shows a moderate positive correlation with BMI (*r* = 0.3), the number of years of experience (*r* = 0.34), and cervical range of motion (r = 0.34). A weak negative correlation is seen with thoracic range of motion (*r* = −0.43). The NDI exhibits a moderate positive correlation with BMI (*r* = 0.24) and a weak positive correlation with the number of years of experience (*r* = 0.2). The NDI shows a strong negative correlation with thoracic range of motion (*r* = −0.65) and a strong positive correlation with cervical range of motion (*r* = 0.84).

To examine how various factors contribute to NPQ and NDI scores, stepwise multiple regression was used (Table 2). The analysis included cervical and thoracic total ROM, years of experience, age, and BMI as potential predictors for NPQ and NDI. The stepwise regression model for NPQ revealed that thoracic total ROM, years of experience, age, and BMI were significant predictors. The model explained 30% of the variance in NPQ scores (*R*^2^ = 0.30, F = 41.09, *p* < 0.01). Specifically, the unstandardized *B* values were −0.40 for thoracic total ROM, 0.82 for years of experience, −0.67 for age, and 0.38 for BMI. For the NDI, the regression model identified cervical total ROM as a significant predictor. This model accounted for 69% of the variance in NDI scores (*R*^2^ = 0.69, *F* = 390.83, *p* < 0.01), with an unstandardized *B* of 0.38 for the cervical total ROM variable.

A mediation analysis was conducted to explore how cervical and thoracic total ROM influence NPQ and NDI scores and examine mediation’s effects. Two mediation pathways were tested: thoracic total ROM as a mediator of cervical ROM’s impact on pain variables and vice versa. Table 3 presents the mediation analysis results. 

Cervical total ROM as a mediator of thoracic ROM’s influence on NPQ showed a significant total effect (*p* < 0.01) with a non-significant direct effect (*p* = 0.58) and a significant indirect effect through cervical ROM (*p* < 0.01), accounting for 80.95% mediation. Thoracic total ROM as a mediator of cervical ROM’s influence on NPQ revealed a significant total effect (*p* < 0.01) and a significant direct effect (*p* < 0.01). Still, the indirect impact through thoracic ROM was insignificant (*p* = 0.58), indicating 10.87% mediation.

Similarly, for the NDI, two mediation pathways were tested. Cervical total ROM as a mediator of thoracic ROM’s influence on NDI revealed a significant total effect (*p* < 0.01), a significant direct effect (*p* < 0.02), but a non-significant indirect effect through cervical ROM (*p* = 0.95), indicating negligible mediation (0.02%). In contrast, thoracic total ROM as a mediator of cervical ROM’s influence on NDI showed a significant total effect (*p* < 0.01), a non-significant direct effect (*p* = 0.95), and a significant indirect effect through thoracic ROM (*p* < 0.01), indicating complete mediation (100%).

## 4. Discussion

The present study highlights a significant association between cervical and thoracic ROM and neck pain severity in young white-collar workers. Specifically, reduced thoracic ROM was linked to higher NP scores, as measured by the NPQ and the NDI. At the same time, an increased cervical ROM was associated with more severe NP symptoms. These findings are supported by previous research and add to the growing body of evidence suggesting that thoracic spine mobility plays a crucial role in the development and persistence of NP.

The relationship between thoracic ROM and NP severity observed in this study aligns with findings from several other investigations. For instance, Edmondston et al. [39] suggested that restricted thoracic mobility could lead to compensatory hypermobility in the cervical spine, increasing strain on cervical structures and exacerbating pain symptoms. Similarly, Tsegay et al. [31] conducted a meta-analysis that found thoracic spine mobilization and manipulation effective in reducing NP and improving cervical ROM, reinforcing that thoracic dysfunction contributes significantly to cervical pain. Our results also resonate with the work of Wang et al. [40], who suggested that the cervicothoracic junction’s biomechanical role is pivotal in maintaining cervical spine stability and function. This is consistent with our findings, where thoracic ROM mediated the influence of cervical ROM on NP, particularly on NDI scores. This mediation indicates that cervical spine mobility’s impact on NP is primarily modulated by thoracic mobility, a relationship that has been echoed by other researchers, including Masaracchio et al. [32] and Joshi et al. [30], who both reported significant reductions in NP following thoracic interventions.

The compensatory mechanisms highlighted in our study are supported by other authors [27,41], who demonstrated that multimodal treatments, including both cervical and thoracic interventions, were more effective in managing NP than treatments focusing solely on the cervical spine. Similarly, a study by Lee et al. [42] identified that limited thoracic ROM could lead to altered cervical kinematics, thereby increasing the risk of developing NP. These findings are consistent with our observation that reduced thoracic ROM is associated with increased NP severity.

Furthermore, the association between thoracic ROM and NP severity has been explored by Cho et al. [43], who found that improving thoracic mobility through specific exercises significantly alleviated NP. This supports our hypothesis that thoracic mobility is critical in preventing compensatory cervical hypermobility and the resulting pain. Additionally, the work of Quek et al. [44] suggested that thoracic spine flexibility is a key factor in maintaining cervical spine health, as it helps distribute mechanical loads more evenly across the spine, reducing the risk of overload in the cervical region. 

The role of posture in NP, particularly the contribution of forward head posture, has been well documented in the literature. For example, Fiebert et al. [14] identified a strong correlation between poor thoracic extension and the development of forward head posture, which increases cervical spine strain and contributes to NP. Our findings are in line with these observations, as participants with reduced thoracic extension exhibited higher cervical ROM and more severe NP symptoms. The interplay between thoracic and cervical ROM is further highlighted by the work of Szeto et al. [45], who reported that individuals with restricted thoracic mobility often develop compensatory cervical hypermobility, leading to increased muscle tension and pain. This is consistent with our findings, where increased cervical ROM was associated with more severe NP, likely due to the increased biomechanical demands placed on the cervical spine in the absence of adequate thoracic mobility. Watson and Trott [46], who found that thoracic spine manipulation effectively reduced NP and improved functional outcomes, also support the significance of thoracic spine mobility in NP management. Similarly, Saal and Saal [47] emphasized the importance of maintaining thoracic spine flexibility to prevent the development of NP, particularly in individuals with sedentary lifestyles. These findings are particularly relevant to our study population, consisting of young white-collar workers at increased risk of developing NP due to prolonged sitting and poor posture.

Moreover, the influence of thoracic ROM on cervical spine health has been explored by Dunning et al. [48], who reported that thoracic-oriented spine treatment led to significant improvements in cervical ROM and pain reduction in patients with NP and headaches. This supports our findings that interventions targeting the thoracic spine could have a beneficial effect on cervical spine function and pain levels. The relationship between thoracic ROM and NP severity observed in our study is further corroborated by a study by Arsh et al. [49], who suggested that patients with reduced thoracic mobility were more likely to experience NP. This highlights the importance of addressing thoracic spine dysfunction in the management of NP, particularly in populations at high risk for chronic pain. It is also vital to highlight biopsychosocial aspects affecting the treatment plan and the clinical outcome, as suggested by Fernandez-Carnero et al. [50] in chronic neck pain patients.

### 4.1. Clinical Implications

The clinical implications of our findings are noteworthy, suggesting that clinicians should integrate thoracic spine evaluations into the standard assessment of patients presenting with neck pain, particularly among young white-collar workers who are at increased risk due to prolonged sedentary behaviors. From a practical standpoint, this indicates that therapeutic interventions should extend beyond the cervical spine to include strategies aimed at enhancing thoracic spine mobility, such as thoracic mobilization techniques and exercises designed to improve thoracic extension. By addressing thoracic dysfunction, clinicians may mitigate compensatory cervical hypermobility, leading to more effective and enduring pain relief. These results underscore the importance of a holistic approach to neck pain management, advocating for comprehensive spinal care that encompasses both cervical and thoracic regions. 

In summary, our findings are consistent with a substantial body of the literature and shed new light on young adults, emphasizing the importance of thoracic spine mobility in developing and managing NP. The observed association between reduced thoracic ROM and increased NP severity underscores the need for a holistic approach to NP management that includes thoracic spine assessments and interventions. Given the complexity of NP and its multifactorial etiology, addressing thoracic spine dysfunction may play a crucial role in preventing and alleviating NP. 

### 4.2. Limitations

Despite the valuable insights gained from this study, several limitations must be acknowledged: First, the cross-sectional design precludes the establishment of causality between thoracic ROM and NP. While the observed associations are significant, the directionality of these relationships cannot be definitively determined, and longitudinal studies are needed to explore causal pathways. Additionally, our target group included individuals with neck pain lasting at least six weeks to exclude temporary pain and focus on more persistent issues without categorizing it as acute or chronic. However, this choice also limits our ability to categorize participants based on pain duration (e.g., ‘chronic’ for over three months or ‘acute’ for less than three months), potentially reducing the depth of insights into the impact of pain duration on outcomes. Third, this study relied on self-reported measures of NP and disability, which may be subject to bias. Although validated instruments such as the NPQ and NDI were used, self-reported data can be influenced by various factors, including psychological state and individual perceptions of pain. Fourth, the study population was limited to young white-collar workers, which may restrict the generalizability of the findings to other demographic groups, such as older adults or those with different occupational backgrounds. Future research should aim to replicate these findings in more diverse populations to enhance their applicability. Finally, this study focused exclusively on sagittal plane movements of the thoracic and cervical spine. Since NP is influenced by movements in multiple planes, future studies should assess ROM in other planes, such as the frontal and transverse, to provide a more comprehensive understanding of the relationship between spinal mobility and NP.

## 5. Conclusions

Addressing thoracic mobility is crucial for effective neck pain (NP) management, especially in young adults. Future research should focus on establishing causal relationships and exploring broader implications in diverse populations. Integrating thoracic spine assessments and interventions into NP management protocols may improve treatment outcomes and reduce NP burden in at-risk populations.

## Figures and Tables

**Table 1:** Participant characteristics.

Variable		Overall	Mild Pain	Moderate Pain	*p* Value
*n*		179	78	101	-
*n* (%) of women		139	58	81	0.35 ^b^
Age, years		30.17 (3.33)	30.06 (3.42)	30.25 (3.28)	0.69 ^a^
Body mass, kg		68.37 (14.89)	65.46 (14.07)	70.61 (15.18)	0.02 ^a^
Body height, m		1.69 (0.07)	1.69 (0.08)	1.69 (0.07)	0.87 ^a^
Working experience, years		7.03 (3.55)	6.08 (3.37)	7.77 (3.53)	<0.01 ^a^
BMI, kg/cm^2^		23.77 (3.86)	22.69 (3.27)	24.60 (4.07)	0.003 ^a^
	Normal (BMI 18.5–24.9), *n* (%)	111 (62.01)	59 (75.64)	52 (51.49)	0.003 ^b^
	Overweight (BMI 25–29.9), n (%)	53 (29.61)	16 (20.51)	37 (36.63)	
	Obese (BMI > 30), *n* (%)	15 (8.38)	3 (3.85)	12 (11.88)	
Self-reported exercise level					
	None, *n* (%)	16 (5.32)	8 (10.26)	8 (7.92)	0.008 ^b^
	Low, *n* (%)	56 (18.60)	19 (24.36)	37 (36.63)	
	Moderate, *n* (%)	91 (30.23)	38 (48.72)	53 (52.48)	
	High, *n* (%)	16 (5.32)	13 (16.67)	3 (2.97)	
Desk regulation, *n* (%)		122 (68.16)	49 (62.82)	73 (72.28)	0.18 ^b^

BMI: body mass index; ^a^ Mann–Whitney *U* test; ^b^ chi-square test.

**Table 2:** Multiple linear regression results (stepwise).

Variable		Unstandardized *B*	Standardized Beta	*t*	*p* Value	*F*	*R* ^2^
NPQ					<0.01	41.09	0.30
	Thoracic total ROM	−0.40	−0.38	−5.63			
	Experience	0.82	0.36	4.56			
	Age	−0.67	−0.28	−3.50			
	BMI	0.38	0.18	2.77			
NDI					<0.01	390.83	0.69
	Cervical total ROM	0.38	0.83	19.77			

NPQ: Northwick Park Neck Pain Questionnaire; ROM: range of motion; NDI: Neck Disability Index; BMI: body mass index.

**Table 3:** Results of the mediation analysis.

Variable		Total Effect		Direct Effect		Indirect Effect		Percentage Mediation
		Effect Size (95% CI)	*p* Value	Effect Size (95% CI)	*p* Value	Effect Size (95% CI)	*p* Value	
NPQ								
	C → Th	0.21 (0.13; 0.28)	<0.01	0.03 (−0.08; 0.14)	0.58	0.17 (0.09; 0.27)	<0.01	80.95
	Th → C	−0.46 (−0.59; −0.33)	<0.01	−0.42 (−0.61; −0.22)	<0.01	−0.05 (−0.20; 0.11)	0.58	10.87
NDI								
	C → Th	0.38 (0.34; 0.42)	<0.01	0.38 (0.32; 0.44)	<0.02	0.001 (−0.04; 0.04)	0.95	0.02
	Th → C	−0.53 (−0.62; −0.45)	<0.01	−0.003 (−0.11; 0.09)	0.95	−0.53 (−0.62; −0.45)	<0.01	100.00

NPQ: Northwick Park Neck Pain Questionnaire; NDI: Neck Disability Index; CI: confidence interval; C: cervical range of motion; Th: thoracic range of motion.

## Data Availability

Data are available upon reasonable request to the corresponding author.
